# Ethics and Equity Challenges in Telerehabilitation for Older Adults: Rapid Review

**DOI:** 10.2196/69660

**Published:** 2025-08-13

**Authors:** Mirella Veras, Louis-Pierre Auger, Jennifer Sigouin, Nahid Gheidari, Michelle LA Nelson, William C Miller, Anne Hudon, Dahlia Kairy

**Affiliations:** 1 Department of Physical Therapy, University of Manitoba, R115-771 McDermot Avenue, Winnipeg, MB, R3E 0T6, Canada, 1 2047893417; 2 The Centre for Interdisciplinary Research in Rehabilitation of Greater Montreal (CRIR), Montreal, QC, Canada; 3 Centre on Aging, University of Manitoba, Winnipeg, MB, Canada; 4 School of Physical and Occupational Therapy, Faculty of Medicine and Health Sciences, McGill University, Montreal, QC, Canada; 5 Clinical Research Unit, Montreal Neurological Institute/Hospital, Montreal, QC, Canada; 6 Lunenfeld-Tanenbaum Research Institute, Sinai Health, Toronto, ON, Canada; 7 Dalla Lana School of Public Health, University of Toronto, Toronto, Canada; 8 Occupational Science & Occupational Therapy, Faculty of Medicine, University of British Columbia, Vancouver, BC, Canada; 9 Centre for Aging SMART (Solutions for Mobility, Activity, Rehabilitation and Technology), Rehabilitation Research Program, Vancouver, BC, Canada; 10 Physiotherapy Program, School of Rehabilitation, Faculty of Medicine, University of Montreal, Montreal, QC, Canada

**Keywords:** aging, older adults, equity, ethics, telerehabilitation, telehealth, review, rehabilitation, digital health, geriatric, virtual care

## Abstract

**Background:**

The integration of technology in rehabilitation is transforming health care delivery for older adults, especially through telerehabilitation, which addresses barriers to in-person care.

**Objective:**

This rapid review explores the ethical and equity concerns associated with telerehabilitation for older adults, focusing on challenges such as internet access, technology adoption, and digital literacy.

**Methods:**

Conducted according to Cochrane Rapid Review guidelines, this review used the Metaverse Equitable Rehabilitation Therapy framework, focusing on equity and ethics. Studies included telerehabilitation services for adults aged 55 years and older, published between 2010 and 2023. Screening was conducted independently by 2 researchers using Rayyan (Qatar Computing Research Institute, Hamad Bin Khalifa University), with full-text review by additional team members. Searches were performed in Medline and CINAHL

**Results:**

From 323 papers retrieved, 49 studies met the inclusion criteria. The included studies were published between 2013 and 2023. Disparities in socioeconomic status, geographic location, and racial and ethnic backgrounds were found to impact telerehabilitation use. Additionally, ethical concerns around privacy, security, and autonomy were often inadequately addressed.

**Conclusions:**

This review emphasizes the need for culturally appropriate, accessible, and inclusive telerehabilitation services that integrate ethical and equity considerations into their design and delivery.

## Introduction

The use of technology in rehabilitation is increasing worldwide, including its application in delivering services to the older adults population [1]. This is transforming how health care services are delivered to the aging population [2]. Telerehabilitation, which involves the evaluation and treatment of patients through technology, has emerged as an attractive option for older adults who may have multiple comorbidities [1,3]. Before the COVID-19 pandemic, numerous adults faced challenges accessing rehabilitation treatments due to diverse clinical limitations and geographic circumstances. Telerehabilitation has assumed greater importance in addressing the needs of older adults, as the COVID-19 pandemic exacerbated difficulties by further restricting face-to-face interactions with implementation of societal measures aimed at lowering disease prevalence [4].

When older adults experience complex medical needs, whether due to multiple chronic conditions or acute illnesses, it can lead to increased dependence on others and diminish the quality of life for both the individuals and their caregivers [5]. Health care technologies can help overcome these challenges and enhance the well-being of the aging population by improving access to rehabilitation [5]. In this context, telerehabilitation provides an alternative platform for delivering health education and care to clients in clinical, community, or home care settings [6]. However, challenges in delivering telerehabilitation to older adults have been reported in the literature and include issues with internet access, adoption of technology, self-efficacy, experience with technology, frequency of usage, and reliance on guidance and digital literacy [7,8]. Other barriers reported in a systematic review about the use of telehealth use among older adults were trust of internet information, reading small fonts, information overload, clicking small icons, interacting with scroll bars, lack of understanding of app capabilities, fear of others overhearing, security, need for support to use, cost, privacy concerns, among others [8].

Despite the numerous benefits of telerehabilitation for older adults, this is recognized as an extremely heterogeneous population; therefore, there is a pressing need to investigate and understand the ethical and equity concerns associated with these services. No reviews to date have thoroughly explored these aspects, which is critical given the reported challenges. Knowing about these problems is important to help address the effectiveness and accessibility of telerehabilitation. Ethical concerns may arise regarding the confidentiality of patient information, while equity issues could stem from disparities in access to technology and the internet among different socioeconomic groups, for example. Addressing these concerns is essential to ensure that telerehabilitation services are delivered fairly and effectively, promoting better health outcomes and quality of life for all older adults. This review aims to examine these concerns and explore telerehabilitation for older adults over the past 10 years.

## Methods

### Study Design

Rapid reviews are a form of synthesis that accelerates the traditional systematic review process, facilitating the dissemination of literature in a resource-efficient manner, particularly relevant in the context of rapidly advancing technology research [9]. This rapid review aimed to explore and summarize the current literature on the equity and ethical concerns associated with delivering telerehabilitation services, following the Cochrane Rapid Review guidelines [9]. To guide the data extraction and analysis of this review, the Metaverse Equitable Rehabilitation Therapy (MERTH) framework [10] was applied, adapted to telerehabilitation, with a focus on equity (accessibility, inclusivity, diversity, fairness, and cultural relevance), health services integration (responsiveness, continuity of care, and autonomy to participate in health-related decisions), interoperability, and humanization (communication, person-centered care, and empowerment) [10]. For this article, we are focusing on equity (accessibility, inclusivity, diversity, fairness, cultural relevance) and empowerment.

We included studies that focused on telerehabilitation services for both older adults and middle-aged individuals aged 55 years and above. The inclusion criteria focused on reviews (systematic reviews, scoping reviews, meta-analyses, and narrative reviews with descriptions of included studies) involving older adults aged 55 years and older, which includes the older working-age population (55 to 64 y) [11]. This age range was selected to include individuals who may benefit from telerehabilitation services, considering their potential health care needs and technological engagement during this phase of life. These studies were selected if published between 2010 and 2023. Editorials, conference abstracts, and papers without full text available were excluded. Two researchers (MV and NG) independently completed the title and abstract screening using Rayyan [12], an online app that accelerates initial screening of abstracts and titles for managing reviews. One reviewer (MV) performed the full-text screening, and any doubts regarding inclusion were discussed with MV, DK, and JS. DK, JS, NG, and MV developed a data extraction tool and refined it throughout the extraction process. For added rigor, a sample of the data, such as 10% (13/125) of the reviews at the abstract phase, was independently reviewed by another team member (DK or JS).

To conduct the search, we consulted a specialized health sciences library. We tailored a search strategy for 2 databases: Medline and CINAHL.

Detailed search strategies can be found in Multimedia Appendix 1.

### Ethical Considerations

This study is a review of existing literature and does not involve any primary data collection or direct interaction with human participants. Therefore, ethical approval and consent for publication are not required. All sources cited have been appropriately referenced per academic standards.

## Results

### Overview

**Figure 1.**
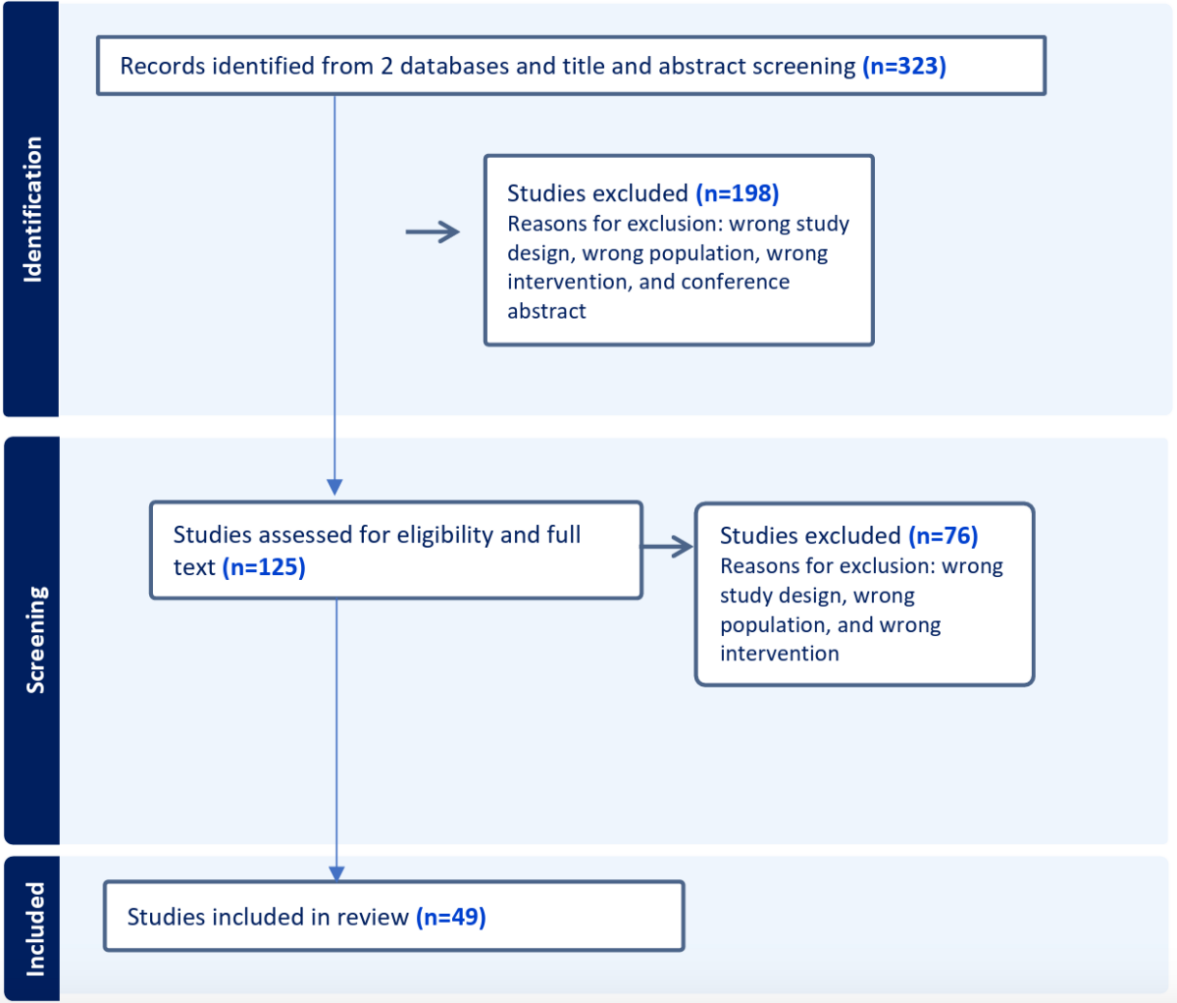
Flow diagram of study selection.

Figure 1 provides an overview of the review process. Initially, 323 papers were retrieved through the search. After title and abstract screening, followed by full-text review, 49 studies met the criteria for extraction and final evaluation.

### Publications Characteristics

The included review studies were published between 2013 and 2023. Approximately 39% (n=19) of the studies were published in 2021. The included studies predominantly consisted of systematic reviews (22 studies), systematic reviews and meta-analyses (11 studies) followed by scoping reviews (8 studies; Table S1 in Multimedia Appendix 2 [6,13-60]).

### Morbidities

Cardiorespiratory, neurological, and musculoskeletal conditions are the primary morbidities present in the studies identified in this review. Other key categories include cognitive impairment and mental health, cancer, and diabetes. Less frequently studied morbidities include vision and hearing problems, balance disorders, risk for falls, and pelvic floor disorders. The distribution of included studies (n=49) across morbidity categories revealed that the most commonly addressed condition was cardiorespiratory, featured in 33/49 (67%) of the studies. Neurological and musculoskeletal conditions followed this, each reported in 17/49 (35%) of the studies. Studies categorized under "other" comprised 15/49 (31%). Cognitive impairment and mental health, and cancer were each represented in 9/49 (19%) of the studies. Diabetes was addressed in 6/49 (13%), while vision and hearing problems, balance disorders and risk for falls, and pelvic floor disorders were the least represented, each occurring in 2/49 (5%) of the included studies. These findings indicate a predominant research focus on cardiorespiratory and neuromusculoskeletal morbidities.

It is important to note that individual morbidities can be counted in more than 1 study within these reviews and that each study could include participants with several morbidities. This repetition highlights the prevalence and importance of specific categories of health issues within the reviewed literature.

### Type of Technologies Used in Telerehabilitation for Older Adults

Within the included reviews, the categorization of communication technologies in telerehabilitation included several key areas, each highlighted by different examples and applications. Communication technologies included eHealth platforms such as telehealth, telemedicine, and telerehabilitation, which used tools such as videoconferencing (Skype [Microsoft Corp] and Zoom [Zoom Communications, Inc]), telephones, text messaging, emails, and web-based platforms. Telemonitoring and remote monitoring were used through health monitoring systems, wearable sensors, and mobile health apps for tracking health metrics. Virtual and augmented reality were applied in digital rehabilitation systems and exergames such as Wii Fit (Nintendo Entertainment Analysis & Development) and Kinect (Microsoft). Wearable technology included sensors, smartwatches, and fitness trackers. Education and support tools provided digital health education via e-learning apps and digital pain coaches. Digital and assistive technologies enhanced patient engagement and supported independent living through interactive internet platforms and emergency assistance systems. Finally, home-based rehabilitation and exercise facilitated remote rehabilitation exercises with personalized programs and activity trackers (Table 1)

Based on the studies included in this rapid review, the most frequent categories of technologies used are communication technologies, including telemonitoring and remote monitoring, which appear in 73% of the included studies. In contrast, the least used categories are virtual and augmented reality and interactive and assistive technologies, each appearing in 12% of the studies. Specifically, virtual and augmented reality were found in 6 studies, which corresponds to approximately 12% of the total studies. The total percentage exceeds 100% due to the overlap of studies across multiple categories (Table 1).

**Table 1.** Study characteristics: technologies used and examples.

Categorization	Examples	References
Communication technologies	eHealth platforms: telehealth interventions, telemedicine, and telerehabilitation platforms Telehealth devices: remote supervision tools, home-health monitoring systems, and teleconsultation platforms Videoconferencing: Skype (Microsoft Corp), Zoom (Zoom Communications, Inc), FaceTime (Apple Inc), Adobe Connect, and Microsoft Teams Telephone and mobile phones: standard calls, smartphones, and telephone support Text messaging and emails: SMS and email Web-based platforms: online multimedia, discussion boards, blogs, and web-based health management portals	[6,13-42,60]
Telemonitoring and remote monitoring	Health monitoring systems: heart health monitoring system, wearable sensors, pulse oximeters, and blood pressure monitors Telemonitoring devices: ECG^a^, heart monitors, Bluetooth-powered sensors, and wireless transmitters Mobile health apps: apps for tracking health metrics and remote wound care applications	[6,13,18,21-23,26,28,30,35,37,39,43-50]
Virtual and augmented reality	Virtual rehabilitation systems: virtual reality–based rehabilitation and interactive virtual reality exercises Exergames: Wii Fit (Nintendo Entertainment Analysis & Development), Kinect (Microsoft), VRc^b^ video game dancing, and interactive gaming systems	[6,19,23,51,52,61]
Wearable technology	Sensors and monitors: inertial sensors, accelerometers, gyroscopes, and pedometers Wearable devices: smartwatches, fitness trackers, and wearable pressure	[6,19,21,41,43-46,53,59]
Education and support tools	Provides health education and support through digital tools. Examples: e-learning apps, digital pain coaches.	[6,14,15,17,21,23,26,30,36,38,45,46,50,54-56]
Interactive and assistive technologies	Enhances patient engagement and supports independent living. Examples: interactive internet platforms, emergency assistance	[17,22,39,50,53]
Home-based rehabilitation and exercise	Facilitates rehabilitation exercises at home with remote guidance. Examples: personalized exercise programs, activity trackers.	[6,15,21,22,24,35,43-45,47,48,50,52,53,55]

a ECG: electrocardiogram.

b VR: virtual reality.

### Equity

#### Sex or Gender

Twenty-nine reviews included at least 1 study that reported findings on gender (Table S1 in Multimedia Appendix 2). The reviews that reported sex or gender in their results did not provide a full analysis based on these variables. However, 2 studies included some comments related to their gender or sex results.

One review reported that the findings regarding gender and the use of eHealth are mixed. “Out of 22 articles, only five suggest that gender plays a role in influencing eHealth usage, while eight studies found no connection between gender and eHealth use.” The studies did not define gender, underscoring the importance of clearly specifying sex, gender, and sexual orientation within the included population. Additionally, 3 studies highlight that women tend to be more engaged with and satisfied by eHealth apps, using them more frequently than men [13]. In 1 review, the authors mentioned a gender bias, with more male participants than female participants [6]. In another study, the authors noted that most of the studies included more women than men. Four studies included only women. One study explained this decision as “to avoid the influence of gender differences on the risk of falling.” Two studies excluded the few male participants, and another study did not provide a reason for the exclusive participation of women. Additionally, 1 study did not describe the age range or gender distribution of the participants [43].

#### Ethnicity

Racial differences in telehealth care use were observed, with cultural minority groups. For instance, 1 study found no significant effect of telehealth interventions on older Hispanic patients with chronic heart failure [14]. Overall, most studies that reported ethnicity included predominantly White participants. The term “Caucasian” is used as a more formal and historically established term to describe people of European descent. However, it can be viewed as outdated or less inclusive compared to simply using “White” or more specific ethnic identifiers (Table S1 in Multimedia Appendix 2).

#### Geographic Location

Older adults with chronic diseases residing in rural areas are less likely to use eHealth services compared to their urban counterparts, primarily due to limited access and lower socioeconomic status. Rural inhabitants face greater barriers to the effective implementation of eHealth, often related to insufficient infrastructure and resources. Contrarily, some regions, such as certain cities in South Korea with limited medical facilities, have shown a higher adoption of eHealth compared to more urbanized areas, highlighting the potential benefits of these technologies in underserved locations [13] (Table S1 in Multimedia Appendix 2).

#### Socioeconomic Status

One study highlighted that despite lower-income groups often facing barriers in accessing eHealth technologies due to economic constraints, their satisfaction levels with using these technologies were found to be comparable to those of higher-income groups once they had access (Reiners et al, 2019) [13]. This suggests that while income initially affects access to eHealth services, it may not impact the perceived benefits and satisfaction derived from using them once accessibility barriers are overcome.

#### Barriers

Digital barriers: the following summaries highlight the main challenges and associated studies for each category concerning the adoption of telerehabilitation among older adults:

Economic barriers: affordability of technology is an obstacle for older adults and those with lower educational backgrounds (Bertolazzi et al, 2024 [57] and Bhattarai and Phillips, 2017 [15]).

Usability challenges: difficulty in using mHealth technologies hinders adoption, exacerbated by lack of exposure (Johnson et al, 2021) [44].

Digital literacy disparities: lower digital literacy among racial or ethnic minorities, low-income groups, and people with sensory and manual dexterity deficits impact technology acceptance (Yi et al, 2021 [58]; Johnson et al, 2021 [44]; Jonker et al, 2020 [45]; Bertolazzi et al, 2024 [57]; Bostrom et al, 2020 [46]; Beckie, 2019 [16]; Gaspar and Lapão, 2021 [43]);

Support from family members: family encouragement aids acceptance and use of eHealth technologies (Jonker et al, 2020) [45].

Technology design and training: user-friendly designs and proper training enhance technology adoption (Johnson et al, 2021 [44] and Bhattarai and Phillips, 2017 [15]).

Inclusion and accessibility: addressing barriers such as income and education levels is crucial for equitable access (Bertolazzi et al, 2024) [57].

Health care provider involvement: clinician support and effective information flow facilitate technology adoption (Bhattarai and Phillips, 2017) [15].

#### Costs

The results related to digital health technology costs are structured into several key categories: costs, benefits, equity considerations, and policy implications. First, the section on cost details the financial implications of adopting digital health technologies, highlighting initial investments, ongoing maintenance costs, and cost analyses. Second, the benefits category outlines the positive impacts of these technologies on telerehabilitation, such as improved patient outcomes, enhanced efficiency, and reduced health care disparities. Third, equity considerations address how digital health technologies can potentially bridge gaps in health care access and outcomes among the older adult population. Lastly, the section on policy implications describes regulatory frameworks, privacy concerns, and recommendations for effective implementation strategies (Table S1 in Multimedia Appendix 2).

### Overview of Costs in Digital Health Technology

#### Background of Costs in Digital Health Technology

Digital health technologies include a range of costs associated with implementation and operation. The following provides a summary of findings concerning cost considerations, supplemented with insights from the studies reviewed. For detailed examples and other comments, examples can be found in Table S1 in Multimedia Appendix 2.

#### General Cost Implications

Telehealth technologies are often highlighted as cost-effective strategies, particularly in reducing hospital and nursing home expenses.

#### Cost-Effective Strategies

For instance, telehealth has been proposed as cost-effective for delivering health education and promoting self-monitoring behaviors among older adults with chronic conditions.

#### Cost Types

Costs in digital health include implementation activities and operational expenses related to eHealth applications.

### Specific Cost and Benefit Types

#### Patient Outcomes

Studies predominantly capture patient outcomes through quality-of-life considerations and physical health status indicators (summary of results of the included studies).

#### Resource Use

Emphasis is placed on reducing home care visits and hospital usage through remote monitoring, aimed at preventing unnecessary hospital admissions (results of included studies).

### Equity and Access Considerations

#### Gender and Socioeconomic Variables

These variables influence the usability and adoption of digital health technologies, highlighting disparities in access and usage (comment on discussion of included studies).

#### Racial and Ethnic Differences

Studies note disparities in telehealth use among different racial and ethnic groups, suggesting cultural acceptance and access barriers.

#### Geographic and Socioeconomic Factors

Access to and effectiveness of telerehabilitation are influenced by factors such as geographic location and socioeconomic status, with rural and lower-income populations facing greater challenges.

### Recommendations and Future Directions Related to Costs

#### Addressing Barriers

Recommendations include designing culture-friendly and accessible telehealth services to enhance engagement among diverse populations.

#### Policy Implications

Policy makers are urged to minimize cost barriers, provide subsidies for technology access, and promote equitable health technology adoption.

### Ethics

#### Overview

Twenty-five studies reported some ethical aspects of the included studies. The following ethical aspects of the included studies can be categorized into safety, adverse events, privacy, empowerment, respect and knowledge of cultural diversity, and inclusion of ethics in research design.

#### Safety and Adverse Events

Safety concerns focus on ensuring that interventions do not cause harm to participants. Studies highlight that most interventions were safe, with no substantial adverse events reported (Huang et al, 2020 [17]; Malaguti et al, 2021 [18]; Johnson et al, 2021 [44]; Kraaijkamp et al, 2021 [19]; Bostrom et al, 2020 [46]; Gaspar and Lapão, 2021 [43]; Reeder et al, 2016 [20]; Solis-Navarro et al, 2022 [31]). The adverse events category pertains to the reporting and management of negative outcomes associated with interventions. Some studies mentioned the importance of feasibility testing to identify potential adverse events (Dennette et al, 2021 [21]; Kraaijkamp et al, 2021 [19]; Devi et al, 2015 [22]; Dequanter et al, 2021 [23]; Ambrens et al, 2022 [24]; Del Pino et al, 2022 [47]; and Ding et al, 2023 [25]).

#### Privacy

Concerns about maintaining the confidentiality and privacy of participant data were noted, emphasizing the need for secure data handling and adherence to privacy regulations (Yi et al, 2021 [58] and Jonker et al, 2020 [45]).

#### Respect and Knowledge of Cultural Diversity

This category highlights the importance of culturally appropriate interventions, especially for Indigenous older adults, and includes the use of culturally relevant content and rewards (Choukou et al, 2021 [26]).

#### Empowerment

In 1 study, the authors highlighted the empowerment divide, noting that despite having access to the necessary hardware and skills to use eHealth apps, some individuals did not use these opportunities because they did not feel personally empowered or believe they would benefit. While the reviewed papers primarily addressed economic and usability divides, the authors emphasized that future research should specifically focus on overcoming the empowerment divide (Reiners et al, 2019 [13]).

#### Inclusion of Ethics in the Design of the Interventions

Several studies highlighted in their discussion the importance of incorporating ethical considerations in the design and implementation of interventions. This includes respecting client preferences, obtaining informed consent, and addressing ethical and privacy concerns (Saito and Izawa, 2021 [27]; Sulz et al, 2021 [28], Su et al, 2020 [29]; Theodoros et al, 2019 [30]; and Tao et al, 2018 [42]).

Our review showed that 26 studies did not address the ethical aspects of their interventions. However, among those that did, several key ethical considerations emerged. Safety was a primary focus, with studies generally reporting no adverse events associated with the interventions (Huang et al, 2020 [17]; Malaguti et al, 2021 [18]; Johnson et al, 2021 [44]; Kraaijkamp et al, 2021 [19]; Bostrom et al, 2020 [46]; Gaspar and Lapão, 2021 [43]; Reeder et al, 2016 [20]; Solis-Navarro et al, 2022 [31]). Adverse event management was emphasized through feasibility testing (Dennette et al, 2021 [21]; Kraaijkamp et al, 2021 [19]; Devi et al, 2015 [22]; Dequanter et al, 2021 [23]; Ambrens et al, 2022 [24]; Del Pino et al, 2022 [47]; Ding et al, 2023 [25]).

Privacy concerns were frequently mentioned, highlighting the need for secure data handling and compliance with privacy regulations (Yi et al, 2021 [58]; Jonker et al, 2020 [45]). Empowerment was also critical, with studies stressing the importance of making participants feel capable and confident in using eHealth technologies (Reiners et al, 2019 [13]). Respect and knowledge of cultural diversity were crucial, particularly for indigenous older adults, calling for culturally appropriate content and rewards (Choukou et al, 2021) [26]. Finally, the inclusion of ethics in research design was recommended, advocating for respect for client preferences, informed consent, and addressing ethical and privacy issues throughout the intervention process (Saito and Izawa, 2021 [27]; Sulz et al, 2021 [28]; Su Jing, 2020 [29]; Theodoros et al, 2019 [30]; and Tao et al, 2018 [42]). These findings emphasize the necessity of integrating comprehensive ethical considerations into digital health interventions to ensure safety, privacy, and respect for all participants.

## Discussion

### Principal Findings

The distribution of morbidities highlights areas where research is concentrated and indicates potential gaps in the literature that warrant further investigation, such as vision and hearing problems, balance disorders, risk of falls, and pelvic floor disorders. This distribution might also reflect conditions that health professionals are more comfortable managing. Therefore, there is a need to develop strategies to offer remote rehabilitation services for these conditions as well.

Older adults have a higher prevalence of chronic obstructive pulmonary disease, a trend that is expected to rise substantially in the coming decades due to aging populations and prolonged exposure to risk factors [62]. It has been estimated that between 55% and 98% of older adults aged 60 years and older have at least 2 chronic diseases, known as multimorbidity. Among these, cardiovascular diseases are the most common [63]. This aligns with our findings that cardiorespiratory issues were the most frequently studied morbidity type.

**Figure 2.**
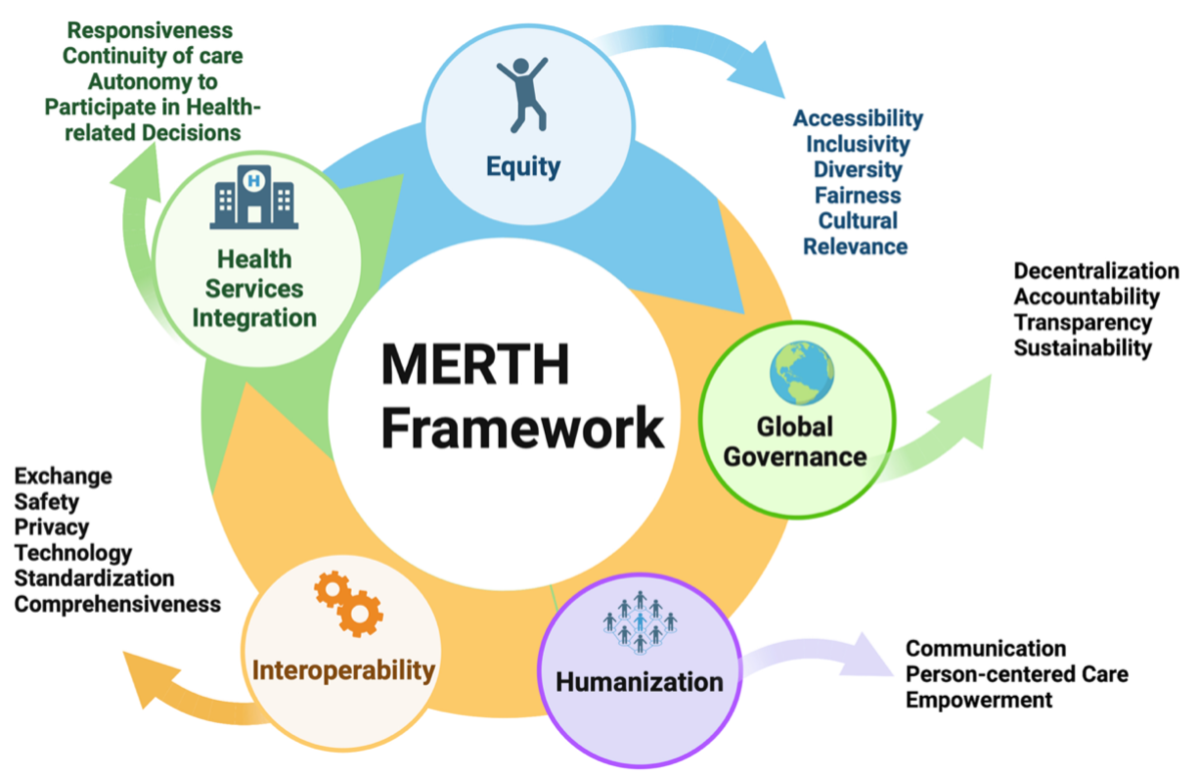
MERTH framework [10]. MERTH: Metaverse Equitable Rehabilitation Therapy.

When considering the Health Services Integration of the MERTH framework (Figure 2), the need for continuity of care and responsiveness becomes even more evident [10]. To effectively address the complex health needs of older adults, particularly those with multiple chronic conditions, health systems must be able to provide integrated and coordinated care across various settings and providers. This not only involves traditional clinical care but also includes social support systems and digital resources, which are increasingly important for managing aging-related health issues [10].

Incorporating telerehabilitation could be a key part of achieving this integration. These digital platforms can help bridge gaps in health care access and ensure that patients receive consistent care, no matter their location or mobility [10]. They also offer an innovative way to enhance autonomy in decision-making, allowing patients to engage in their rehabilitation process more actively and with greater flexibility. This is especially important as older adults may have difficulties attending in-person sessions due to transportation issues or other physical limitations.

In the rapid review, communication technologies, telemonitoring, and remote monitoring were the most frequently included categories, totaling 38 reviews. In contrast, virtual and augmented reality, as well as interactive and assistive technologies, were less frequently cited, with only 6 reviews each. This distribution indicates a stronger focus on technologies that enable direct communication and continuous health monitoring, while immersive and interactive technologies, though valuable, are less commonly studied.

This review highlights several key factors influencing the use and effectiveness of telerehabilitation services across different demographics. Racial disparities are evident, with cultural minority groups often showing lower acceptance of telehealth. For instance, 1 study found no significant impact of telehealth interventions on older Hispanic patients with chronic heart failure, indicating that cultural differences may influence outcomes. There is a substantial research gap in addressing racial differences in telerehabilitation, and the current literature primarily identifies barriers without proposing solutions to promote racial equity in telerehabilitation use. To ensure successful interventions, health care providers must design and deliver culturally friendly telerehabilitation services tailored to their target patients. Future research should focus on exploring these racial differences further and developing strategies to enhance the acceptance and effectiveness of telerehabilitation among diverse racial and ethnic groups.

Geographic location also plays a crucial role, with older adults in rural areas less likely to use telerehabilitation services due to limited access and lower socioeconomic status. These rural barriers often stem from insufficient infrastructure and resources, although some regions, such as certain South Korean cities with limited medical facilities, have shown higher eHealth adoption, demonstrating the potential of these technologies in underserved areas [13]. The overall trend emphasizes a persistent urban-rural divide in telerehabilitation usage, necessitating targeted efforts to improve access and infrastructure in rural areas to ensure equitable health care delivery.

Socioeconomic status further influences telerehabilitation use; lower-income groups face initial access barriers but report satisfaction levels comparable to higher-income groups once they gain access, indicating that economic constraints primarily affect initial accessibility rather than ongoing satisfaction and perceived benefits. The study on gender disparities in telehealth use among older adults in the United States during the COVID-19 pandemic provides relevant results for comparison with our review. It showed a significant increase in telehealth use among females during the pandemic, influenced by factors such as multimorbidity, tablet ownership, and learning new technologies. Males with similar factors also had higher odds of telehealth use [64]. Although some reviews in our study mentioned gender differences, they did not provide much detailed information. These findings emphasize the necessity for targeted interventions to enhance older adults’ access to telerehabilitation services and mitigate digital disparities, aligning with our review’s emphasis on the need for equitable access across diverse populations.

The reviews included in this analysis did not provide a detailed discussion on gender, sexual orientation, or the distinction between sex and gender. While the term “gender” may have been used in some instances, it was not consistently defined or explored in the context of the studies reviewed. In fact, many of the reviews primarily focused on sex-based differences, and in some cases, the term “gender” may have been used interchangeably with sex without clarifying the distinction. Additionally, there was no substantial examination of sexual orientation, and these factors were not adequately addressed or analyzed across the included studies. To provide a more comprehensive understanding of how these variables influence telerehabilitation outcomes, future research should ensure a clear and explicit discussion of gender, sex, and sexual orientation, and their potential impact on the accessibility and effectiveness of telerehabilitation services.

Digital literacy plays a significant role in the adoption of telerehabilitation among older adults, with several key challenges highlighted in this review. Economic barriers, such as the affordability of technology, pose significant obstacles, particularly for older adults and those with lower educational backgrounds. Usability challenges, often exacerbated by a lack of exposure to health technologies, further hinder adoption. Studies show that enhancing internet use among older adults can significantly improve their access to health information and their ability to manage their health [65,66].

Disparities in digital literacy are particularly pronounced among racial or ethnic minorities, low-income groups, and individuals with sensory and manual dexterity deficits, affecting technology acceptance and use. Family support has been shown to aid in the acceptance and use of health technologies, while user-friendly design and proper training can significantly enhance adoption rates. A randomized controlled trial on technophilia (enthusiasm toward new technologies) among people with dementia or mild cognitive impairment and their caregivers demonstrated technophilia was lower among persons with disabilities influenced by factors such as younger age, male gender, higher education, better health, and depression. For caregivers, lower burden and better quality of life also contributed [67]. The sensory and manual dexterity deficits can significantly impact how individuals accept and use technology. These challenges highlight the importance of designing inclusive technology solutions that accommodate a diverse range of physical abilities, ensuring equitable access and usability for all users. Addressing all these barriers is crucial for ensuring equitable access to these technologies.

In terms of accountability, as per the MERTH framework, telerehabilitation services must maintain high standards of care despite these barriers. One area where accountability becomes particularly important is in ensuring that health care providers are properly trained and equipped to offer culturally competent and accessible digital care [10]. As there is a disparity in the acceptance and effectiveness of telerehabilitation across different demographics—such as rural versus urban populations, and racial or ethnic minorities—health care providers must be held accountable for creating inclusive services. This includes adapting technologies to be more accessible, as well as implementing feedback mechanisms to assess the effectiveness of interventions.

Cost considerations include initial investments and ongoing maintenance, but digital health technologies are generally seen as cost-effective, particularly in reducing hospital and nursing home expenses [14,68,69]. To maximize benefits and ensure equity, it is important to design culturally friendly and accessible telerehabilitation services, minimize cost barriers, and promote equitable technology adoption across diverse populations [14]. These findings support our review results on the need for targeted interventions to improve access to telerehabilitation services and reduce digital disparities among older adults.

Regarding ethical aspects, 26 studies did not address the ethical aspects of their interventions. This gap in addressing ethical considerations highlights a substantial shortcoming in the research, underscoring the need for more rigorous ethical evaluation in future studies. However, for the studies that did address ethics, several key considerations came to light. Safety was reported as a comment or result with no significant adverse events associated with the interventions. Adverse event management was emphasized through feasibility testing. A recent scoping review of risks, adverse effects, and mitigation strategies in delivering mental health services via telehealth, emphasizes the growing recognition of the importance of adverse event reporting in telehealth. However, it also highlights the lack of standardized protocols for this purpose [61]. Challenges persist in the telerehabilitation regarding the systematic collection and evaluation of adverse events. The study urges improved reporting of near-misses and actual incidents to enhance risk management. It also advocates for comprehensive clinical training to prevent adverse events and proposes the establishment of robust reporting mechanisms to promote ongoing enhancements in telehealth practices [61]. There is a clear and pressing need to establish standardized protocols for reporting adverse events in telerehabilitation, as highlighted by the findings. This will enable better risk management and improve the overall quality and safety of telerehabilitation services.

The review authors emphasized the importance of addressing privacy concerns and noted a lack of attention to these issues in the included studies. This highlights the need for secure data handling and adherence to privacy regulations. The studies reviewed here briefly addressed privacy and security concerns but did not thoroughly investigate these aspects. Privacy risks in telerehabilitation practice include environmental factors (such as inadequate private spaces for sensitive consultations), technology issues (including data security and limited internet access), and operational challenges [70]. The privacy concerns raised in the review align closely with elements of the MERTH framework: interoperability, particularly regarding exchange, safety, privacy, technology standardization, and comprehensiveness [10]. The challenges highlighted, such as inadequate private spaces, data security issues, and limited internet access are foundational to the safe and effective exchange of information in telerehabilitation. These issues underscore the importance of prioritizing robust data handling and adherence to privacy regulations to safeguard sensitive information of older adults [10].

Empowerment was also critical, with studies stressing the importance of making participants feel capable and confident in using health technologies. There is a noticeable gap between the digital technologies developed for older adults and what they actually need. Aging is often viewed negatively, with older adults stereotypically seen as frail and incompetent [20]. Consequently, many technologies designed for this demographic focus primarily on care rather than empowerment. These negative stereotypes commonly result in excluding older adults from the research and design processes of digital technology. Mannheim et al [71] argue that including older adults in these processes is crucial for creating technologies that genuinely enhance their well-being. Age limits are frequently justified by ageist assumptions that question the competence and reliability of older adults. Social exclusion, driven by these stereotypes, is evident in various areas, including access to services, social relations, and research participation. Arbitrary upper age limits, which more than half of the relevant studies impose, often result in the unjustified exclusion of older adults from clinical research and randomized controlled trials. Although the use of these exclusion criteria may be declining, selective exclusion remains common [71]. These practices highlight ethical challenges in recognizing older adults’ ability to participate meaningfully in research and design, underscoring the impact of stereotypes on their involvement.

This issue aligns closely with the MERTH framework’s element humanization, which emphasizes the importance of person-centered care, communication, and empowerment in health care [10]. The humanization of health care, especially in the context of telerehabilitation, means creating environments where patients are treated as whole individuals, not just as a collection of symptoms or conditions. This approach fosters respect, dignity, and empowerment, encouraging patients to engage actively in their own care and decision-making. In the case of telerehabilitation and digital health technologies, this translates to providing digital spaces that not only cater to physical rehabilitation but also consider emotional, social, and psychological needs [10].

Respect and knowledge of cultural diversity were crucial, particularly for Indigenous older adults, calling for culturally appropriate content and rewards [26]. There is a lack of studies that include Indigenous older adult populations. A study conducted with Indigenous older adults in Saskatchewan showed that many express a desire to learn more about technology and recognize its value in supporting healthy aging [26]. The study highlighted that some researchers emphasized the importance of Indigenous language apps and the revitalization of cultural teachings, seeking technology’s assistance in facilitating these cultural learning processes. It emphasizes the importance of considering the role of families in engaging older adults with technology, given the crucial cultural role families play within Indigenous communities [26].

The findings of this review underline the necessity of integrating comprehensive ethical considerations into telerehabilitation interventions to ensure safety, privacy, and respect for all populations.

### Study Strengths and Limitations

The strengths of this rapid review include its comprehensive synthesis of a wide range of literature within a condensed timeframe, providing timely insights into telerehabilitation for older adults. This efficiency allows for a quick overview of key findings and trends, aiding health care practitioners and policy makers in making informed decisions promptly. Additionally, the review’s focus on ethics and equity in telerehabilitation ensures that critical considerations around patient safety, privacy, and inclusivity are highlighted, promoting ethical standards in telerehabilitation delivery. Finally, by emphasizing the integration of diverse perspectives and the identification of gaps in current research, this rapid review contributes to advancing knowledge and guiding future investigations aimed at enhancing telerehabilitation effectiveness and accessibility for older adults.

Study limitations include language constraints, as searches were limited to English and French publications. Future research should explore strategies to access studies in all languages, particularly in equity contexts, to ensure broader inclusivity. Additionally, the review did not ascertain the specific rehabilitation professionals involved in the studies, as individual study details were not accessed, and this information was, for the most part, not presented in the reviews. Furthermore, data extraction was conducted by a single reviewer, which may have introduced potential biases. To address this, the single reviewer discussed the results with the team and engaged in iterative discussions to mitigate any potential biases. Additionally, to further ensure accuracy, 10% of the reviews, including abstracts, were independently cross-checked by another team member. These limitations highlight the need for comprehensive approaches in future reviews to enhance inclusivity and rigor in synthesizing evidence across diverse contexts and disciplines.

### Conclusions

In conclusion, this review has shed light on the distribution of studies across various health conditions within telerehabilitation contexts, emphasizing a predominant focus on cardiorespiratory, neurological, and musculoskeletal conditions. These findings highlight both the areas of concentrated research and gaps, particularly in less-studied morbidities such as vision and hearing problems, balance disorders, and pelvic floor disorders.

The review also emphasizes disparities in technology adoption and research participation among different demographic groups among older and middle-aged adults, particularly highlighting racial disparities and socioeconomic influences on telerehabilitation use. Challenges persist in addressing these disparities, with rural areas facing barriers due to limited infrastructure and resources. Moreover, the review has identified ethical considerations as paramount, emphasizing the need for standardized protocols in adverse event reporting, improved privacy, and inclusive research practices that recognize and respect cultural diversity, including Indigenous older adults. Moving forward, addressing these gaps and challenges is essential to advancing equitable access to effective telerehabilitation services for older adults across diverse populations.

## Supplementary material

10.2196/69660 Multimedia Appendix 1 Search strategy for this paper.

10.2196/69660 Multimedia Appendix 2 Study characteristics of included studies.

## Abbreviations

MERTH: Metaverse Equitable Rehabilitation Therapy
