# Supplementary material for: Ethics and Equity Challenges in Telerehabilitation for Older Adults: Rapid Review
Source: JMIR Aging. 2025 Aug 13;8:e69660. doi: 10.2196/69660 (PMC12349735; doi:10.2196/69660)
Supplement: Multimedia Appendix 1 [file aging-v8-e69660-s001.docx]

| Table S1. Study characteristics of included studies. | | | | |
| --- | --- | --- | --- | --- |
| f | Study Author  Year [reference]  Study Design  N# included studies | Participants  Morbidity  /Population  Gender | Ethical principles  Comments | Equity aspects  Comments |
| 1 | Saito and Izawa (2021)[27]  SR  N=6 | Heart diseases, pulmonary disease cancer, stroke  **Gender**  Reported (males and females included) | “The appropriateness of adapting Home-based telerehabilitation intervention should be judged by health professionals taking into account broad aspects such as effectiveness, feasibility, client preference and ethical and privacy issues.” | “Moreover, gender and socioeconomic variables can influence the usability of digital health technology. “ (comment discussion) |
| 2 | Sulz et al (2021)[28]  Scoping Review  N=55 | Cardiovascular problems, chronic obstructive pulmonary disease, a chronic skin problem, diabetes, age-related macular degeneration, post-knee arthroplasty patients, Parkinson disease, and terminal patients. Five studies did not specify any health conditions or diseases, mental and behavioral disorders such as anxiety, dementia and depression  **Gender**  NR | “In this sense, the eHealth application is considered a static technology. In the longer run, however, the eHealth application might require an upgrade to comply with new laws and regulations, for reasons pertaining to data privacy, data security, data storage, or improvements in usability.” | “ Some cost and benefit types have received more attention than others. For instance, patient outcomes have been predominantly captured via quality-of-life considerations and various types of physical health status indicators. From the perspective of resource usage, a strong emphasis has been placed on home care visits and hospital usage.” (results of included studies)  “The identified papers considered a range of different types of costs and benefits. Costs pertain to implementation activities and operating activities related to eHealth applications. Benefits (or consequences) can be categorized according to stakeholder groups, that is, patients, caregivers, and health care provider organizations. These benefits can be further divided into stakeholder-specific outcomes and resource utilization. Some cost and benefit types have received more attention than others. For instance, patient outcomes are predominantly captured via quality-of-life considerations and various types of physical health status indicators. From a resource utilization perspective, a strong emphasis is placed on home care visits and hospital usage. One reason for this emphasis is the frequency in which studies focus on remote monitoring to prevent unnecessary hospital admissions or to substitute for home care visits.” (results of included studies) |
| 3 | Guo and Albright (2018)[14]  scoping review of literature  N=31 | Patients with chronic condition, including general chronic conditions, chronic heart failure, diabetes, chronic pain, chronic obstructive pulmonary diseases, chronic lung diseases, hypertension, rheumatoid arthritis, overactive bladder, and chronic anti-vitamin K antagonists.  **Gender**  NR | NR | **Cost**  “Telehealth technologies have been identified as cost-effective strategies to significantly reduce the medical expense of hospital and nursing home stays.” (comment introduction)  “Based on the findings, we propose that telehealth is a cost-effective strategy to deliver health education to promote self-monitoring behaviours to older adults with chronic conditions.“ (comment on discussion)  **Race/ethnicity**  “it is critical to consider racial diversity and culture-related health behaviour differences when using telehealth technologies.” (comment on discussion)  “Racial differences in telehealth care utilization were observed. For example, one study reported that telehealth intervention has no significant effect on older Hispanic patients with chronic heart failure… Due to the different cultures of telehealth recipients, cultural minority groups might lack acceptance of telehealth, a new way of health-care delivery, which could be a key barrier to tele- health’s effectiveness. “ (comment on discussion)  “There is big research gap in the current literature to study racial differences on tele- health care utilization. Even though our review identified racial differences, the current literature failed to move beyond barrier identification toward recognizing solutions that can promote racial equality on telehealth care use.” (comment on discussion)  “We propose researchers further explore racial differences on telehealth utilization and health-care providers design and deliver culture-friendly telehealth services to their target patients for a successful intervention.” (comment on discussion)  “Using telehealth technologies is an advisable intervention for promoting older patients’ self-management. However, it is critical to consider racial diversity and culture-related health behaviour differences when using telehealth technologies.” (comment on discussion)  “In addition, racial differences in telehealth care utilization were observed. For example, one study reported that telehealth intervention has no significant effect on older Hispanic patients with chronic heart failure.” (results) |
| 4 | Dennette et al (2021)  [21] Mixed Method review  N=7 | COPD and cardiovascular disease, asthma, coronary heart disease, diabetes, atrial fibrillation, heart failure, hypertension, stroke/ transient Ischaemic attack, lung cancer or osteoporosis, MSK, cardiovascular disease, diabetes, osteopenia/osteoporosis, and depression, diabetes, congestive heart failure and/or ischaemic heart disease, sleep-disordered breathing and osteoporosis  **Gender**  Reported (males and females included) | Adverse events outcomes included, but not reported. | “ There is a substantial health burden for people living with COPD and multimorbidities, with associated cost implications due to an increased need for hospital utilisation compared to those who only have one condition.” (comment introduction) |
| 5 | Su et al (2020)[29]  SR & MA  N=14 | Coronary heart disease  **Gender**  NR | “To enable peer support through health technology, further eHealth Cardiorespiratory interventions may need to protect the privacy of the participants for peer interaction and provide credible and relevant sources for peer discussion, encourage peer communication and sup- port, role model positive experiences and find resolutions for problems and barriers (Ware et al., 2017).” | **Access**  “ Most reviewed studies were conducted in developed countries, which recruited participants with previous cardiorespiratory experiences.” (comment on discussion, related to the results of the included studies) |
| 6 | Theodoros et al (2019)[30]  Systematic scoping review  N=1 | Parkinson's disease **Gender**  NR | “Similarly, Dorsey et al. (2016) highlighted the shortcomings of current PD care in relation to safety, efficiency, effectiveness, patient-centeredness and equity. “ | **Cost**  Two included studies assessed costs (comment on results)  “The major barriers to accessing speech pathology services include geographical location, avail- ability of clinicians with PD-specific expertise, the motor and psychological symptoms of PD, and cost.” (comment on results) |
| 7 | Tao et al (2018)[42]  SR  N=14 | Hearing problems  **Gender**  NR | “…more research is required to determine overall service quality equivalency to face-to-face service, and to clarify benefit versus harm.”  “Clinicians should consider all options in their decision-making and be alert to new published evidence that clarifies the balance of benefit versus harm.” | **Cost**  “There is a lack of research on the cost-effectiveness of tele-audiology services.” (comment introduction) |
| 8 | Reiners et al (2019)[13]  Literature review  N=22 | Chronic diseases  **Gender**  The results regarding gender are not consistent. Only five out of 22 articles studies show that gender is a factor influencing the use of eHealth whereas in eight studies no relation is found between gender and the use of eHealth. In three studies, women are found to be more engaged and satisfied with eHealth applications and use them more often than men. In contrast, the studies by Terschüren et al. and Kamis et al. show men to be more likely to accept telemonitoring than women.” | **Empowerment**  The third and final form is the empowerment divide, which related to the fact that although they have access to the right hardware, and they have the skills to use the eHealth applications, some people will not make use of the opportunities offered by eHealth, because they do not feel personally empowered to do so and do not think they will benefit. The empowerment divide is much more complicated to overcome. In their suggestions for interventions, the articles included in this literature review therefore mostly address the economic and the usability divide. Therefore, in future research, the empowerment divide deserves specific attention. “ | **Ethnicity/culture**  “ Four articles report no correlation between eHealth use and ethnicity. Whittemore et al. indicated that black, Hispanic, or mixed race/ethnicity are more likely to refuse to enroll in Internet-based research compared to white adolescents. Also, it was suggested that non-white participants experienced pressure of the healthcare provider to enroll. Once they were enrolled in the research though, the satisfaction was as high as for white participants. Suggested reasons for refusal are problems with Internet access or financial issues that lead to a lack of Internet availability. Nelson et al. found that race is significantly related to text responses in eHealth. Non-white participants are less likely to respond to text messages compared to white participants. Similar results were found for participation in interactive voice response calls.” (results)  “eHealth should also be tailored to cultural attitudes and beliefs to increase engagement.” (comment on results)  **Geographic area**  Six articles reported that people with chronic diseases living in rural areas are less likely to use eHealth compared to urban citizens. They were found to have less access to eHealth, and the implementation of eHealth is less effective. As assessed by Duplaga et al. the use of eHealth is more common in urban areas with more than 100,000 inhabitants. As explained by Han et al., the geographical influence is often related to lower socioeconomic status. A positive effect of eHealth was seen in South Korea. A city with few medical facilities showed greater use of eHealth compared to an urban city. Contrarily, Terschüren et al. showed that the place of residence does not play a role in the use of telemedical devices among people with chronic diseases.” (results)  **SES**  “Five articles addressed the relation between the income of the patients and the use of eHealth. People with higher income tended to have more interest in eHealth compared to people with lower income. It is indicated that lower income is associated with limited availability and access to Internet healthcare resources. On the other hand, Whittemore et al. found that in people with lower income, the satisfaction in using eHealth is as high as for people with higher income. In the studies of Nelson et al. and Song et al. income was not a factor influencing the engagement in eHealth.” (results)  **Education**  Three studies indicate that education is not associated with the use of eHealth. However, six studies indicate that education does play a role in the use of eHealth in chronic disease. These articles show that higher education is correlated with more knowledge as well as more use of eHealth technologies. People who are educated to a higher level are more interested and more experienced in using eHealth. Also, a lower level of education is associated with less adherence to telehealth.” (results)  “Several studies show that lower educated people and older people need more support for being able to use eHealth, because they experience more problems with accessing and understanding eHealth information.” (results)  **Culture**  “eHealth should also be tailored to cultural attitudes and beliefs to increase engagement.” (comment discussion)  **Cost/ income**  “Mobile phones are common even among lower income populations and are cost effective.” (results)  “Older people prefer using computers compared to mobile phones, and therefore using the mobile phone as the only option still leads to disparities in eHealth.” (Discussion on results)  “Other suggestions are to subsidize the costs of computer devices, as technology is found to be costly.” (comment on results)  **Digital Literacy**  “Family members can also help patients to accept eHealth by encouraging them, especially helping them in case of low eHealth literacy.” (comment on results)  “Older people and lower educated people need more support when using eHealth technologies.“ (comment to results)  “First, there is the economic divide, manifested in the fact that not everyone can afford to buy the hardware needed to access eHealth. This is influenced by factors such as income, vocational status and place of residence. Second is the so-called usability divide, meaning that some people may not be able to achieve the benefits of eHealth because it is too difficult for them to understand and use effectively.” (discussion)  **Access to technology/ Income**  “People not owning any technical devices to access health information should not be excluded.” (comment on results)  “Access to Internet should be facilitated and made as easy as possible. For instance, for low-income groups, it is suggested that mobile phones should be used, as communicating using a text message is easy and cheap. Others argue that communicating by making a phone call is a better option, because making a phone call does not require literacy skills and might be suitable for less educated.” (comment results) |
| 9 | Tschoepe et al [32]  (2022)  SR  N=27 | Traumatic Spinal Cord Injury  **Gender**  Reported (all included studies had more than 65% of male participants) | NR | **Cost**  “High medical and psychosocial costs during the first year after injury suggest that traditional medical models may fall short in addressing the needs of individuals with new tSCIs as they make the initial transition from acute care to community settings. This complex transition has been described as one of the most difficult aspects of life after SCI.9 In response to increased care costs, shorter hospital lengths of stay, and an acknowledgment that lifelong follow-up is critical, care providers and research groups have responded with novel approaches to transition care.” (comment introduction) |
| 10 | Van Egmond et al (2018)[33]  SR & MA  N=23 | Post cardiac, abdominal, thoracic, cervical, oncological and orthopaedic surgery  **Gender**  Reported (around 56% females in the included studies) | NR | **NR** |
| 11 | Velayati et al (2020)  [51]  SR  N=8 | Stroke, chronic obstructive pulmonary disease (COPD), total knee replacement, and in patients with the comorbidity of COPD and chronic heart failure.  **Gender**  NR | NR | **Cost**  **“**In stroke patients, telerehabilitation helped to improve their ability to do daily works and reduced costs and the duration of rehabilitation programs.” (comment introduction) |
| 12 | Whitfield et al (2022)  [34]  SR & MA  N=10 | Major depressive disorder; primary anxiety and/or mood disorder; or multiple sclerosis  **Gender**  (the proportion that were female ranged from 48% to 88% (median 75% female) | NR | **Cost**   “No cost-effectiveness data were available in this (or any) study.“ (comment on results) |
| 13 | Yi et al (2021)[58]  SR  N=17 | Dementia  **Gender**  (One included study reported gender, both male and female included) | **Privacy**  “Patients partners indicated they did not have concerns about privacy.” | **Education and Socioeconomic status (SES)**  **“**Among studies reporting race and ethnicity, education level, or socioeconomic position, participants were predominantly self-identified as white and were educated individuals from higher socioeconomic backgrounds familiar with using technology. “ (comment on results)  **Digital Literacy/Ethnicity/SES**  “No studies accounted for how factors that are known drivers of technology acceptance among older adults, such as eHealth literacy, technology self-efficacy, and preferences and prior experiences, may influence the feasibility and acceptability of telemedicine. Given that racial/ethnic minority and low-income adults have lower rates of high-tech device use.” (comment on results) |
| 14 | Huang et al (2020)[17]  SR & MA  N=13 | Cancer  **Gender**  Authors reported that gender was extracted, but did not present the results | **Safety**  “Medical staffs can conveniently guide cancer patients to conduct self-management training by Internet, and they could observe patients’ treatments from time to time, such as the intensity of exercise and could pay close attention to patients’ reaction during the intervention to ensure patient safety.” | **Ethnicity**  Ethnicity mostly reported (China), authors stated they extracted gender, but they didn't report it in the review. This study obtained studies from several different countries. The physical fitness and ethnicity of the subjects in this literature were enormously different, so the results might accompany with some clinical heterogeneity.” (comment on results) |
| 15 | Malaguti et al (2021)  [18]  SR & MA  N=21 | Chronic obstructive pulmonary disease  **Gender**  (17 studies reported gender (most of the studies that reported gender had more males than females. In general more male participants in the included studies). | **Safety**  “There were no adverse events related to maintenance programmes, suggesting that maintenance programmes are safe.”  “ No adverse events were reported.” | **Cost**  “ The costs of delivering maintenance programmes are likely to vary across different models, but these costs have not been well documented and the impact of maintenance programmes on other healthcare costs (e.g. hospitalisation, primary care visits) is not totally known. One previous systematic review and meta‐analysis showed that supervised maintenance exercise was effective in reducing the rate of respiratory cause hospital admissions (Jenkins 2018). However, other outcomes such as direct care costs during the follow‐up period, adverse events, exercise capacity and quality of life were not reported.” (comments on discussion)  “ It is uncertain whether there would be benefits to the health system, such as reduced costs related to hospitalisation and also the commissioning of maintenance programmes after pulmonary rehabilitation.” (conclusion) |
| 16 | Johnson et al. (2021) [44]  Narrative review  N=8 | Osteoarthritis  **Gender**  (one study reported unbalanced gender (19 women and 2 men)) | **Safety**  “More RCTs and pragmatic clinical trials are needed to gain a better understanding of the usability, feasibility, efficacy, effectiveness and safety of smart technologies and their integration into routine care.” | **Gender**  “Unbalanced gender representation in one study: 19 women and 2 men. (results)  **Digital literacy**  “The authors noted that a significant number of older adults lacked prior exposure to mHealth technology, but this barrier could be overcome with access and exposure to mHealth devices via community-based programmes. wearable smart devices (wearable smartphones and tablets) might be cost prohibitive or simply inaccessible for some patients, which could exacerbate health-care disparities.” (discussion)  “Designers of smart technology should include easy communication between the individual and the technology. The nature and content of the training might also be a barrier to use of smart technology (time required to learn to use the device, how to use, how to interpret.” (Discussion) |
| 17 | Ollevier et al (2020)[53]  SR  N=7 | Healthy older adults  **Gender**  (One study included only women, other studies had on average 67% women and one study did not mention gender) | NR | **Education**  “Various education levels were reported, an average of 30% participants had a high school education level or less, 50% had an education level between an associate and a bachelor's degree and 64.8% had limited formal education. “ (results) |
| 18 | Kraaijkamp et al (2021)[19]  SR  N=40 | Stroke, orthopedic and cardiac problems and multiples diagnoses (not listed in the article)  **Gender**  (Percentage of female varied between 16 % to 90%. ) | **Adverse events**  “To better understand how eHealth can be used safely, feasibility testing is an important first step. The aim of feasibility testing is to “determine whether an intervention is appropriate for further testing”, but a general accepted standard on feasibility testing is lacking. Examples of factors that can be addressed in feasibility testing are adverse events, adherence, and acceptability.”  “ A combination of these age-associated conditions triggers an increased risk of adverse outcomes such as hospitalization, functional impairments, and even mortality.”  “ 6 studies (30%) reported outcomes related to “adverse events.”  “ None of the included studies reported serious adverse events during the study period [41,46,50,51,74,76]. However, 2 studies (of 40, 5%) reported that some participants experienced discomfort during exergames.” | **Cost**  “ 2 studies (40%) included robotics as interventions and found significant differences in cost, in favor of the intervention group.”(results)  “Hesse and colleagues and Vanoglio and colleagues reported decreases in cost with the use of robotics in comparison with either regular arm therapy (€4.15 [US $4.92] for robotic interventions vs €10.00 [US $11.85] for regular arm therapy, for each patient per session) [42] or physiotherapy (€237 [US $280.73] for robotic intervention vs €480 [US $568.57] for physiotherapy, for each patient per 30 days). In contrast, Schoone and colleagues reported an increase in total costs when compared with physiotherapy (€644.14 [US $762.99] for robotic interventions vs €423.74 [US $501.93] for physiotherapy).”(results) |
| 19 | Jonker et al (2020)[45]  SR  N=7 | Lung cancer, cardiac surgery, radical cystectomy, Joint arthroplasty and Surgery for esophago-gastric cancer and Carotid end-arterectomy for carotid stenosis  **Gender**  Not reported | **Privacy**  “ Future studies should focus on the possible barriers to implementation of perioperative eHealth interventions, such as provider and patient satisfaction, absence of regulations concerning safety and privacy of eHealth platforms,[…]” | **Digital Literacy/Disability**  “Reasons reported for exclusion of patients were related to type of surgery or disease, insufficient understanding of the required language, no Internet or smartphone, and inability to provide consent. Lowres et al. reported exclusion of 4 of 131 patients with impaired cognition, 1 because of impaired vision, and 2 because of mental illness. “(results)  **Education**  “Patient characteristics such as functional status and level of education were reported in only 2 of 7 studies. Of the 44 participants in Lowres et al., (45%) did not complete high school. Granger et al. reported that >60% of their patients had a high performance status.”(results)  **Socioeconomic status**  “Regarding socioeconomic status, authors mentioned that most patients lived at home with family or support. Other studies did not report patients’ functional status, level of education, or socioeconomic status.”(results)  **Digital Literacy**  “Future studies should focus on the possible barriers to implementation of perioperative eHealth interventions […] and exclusion of “patients with low digital literacy. “ (recommendation) |
| 20 | Linn et al (2021)[48]  Scoping review  N=105 | Parkinson’s’ disease  **Gender**  (5 included studies reported gender and males and females were included) | **Adverse outcomes**  Two studies investigated adverse outcomes | **Cost**  Cost analysis reported in 7 studies (7%) (results)  “we highlighted that its efficiency or cost-effectiveness can be further reviewed in future research.” (comment on discussion)  “We believe it is essential to gather stronger evidence that new technologies are delivering the desired results and to balance those benefits with the risks and costs.”(conclusion) |
| 21 | Johnsen et al (2021)[59]  SR  N=15 | Back and neck pain, mental health, chronic MSK disease, cancer, gynecological surgery, fatigue, depression, abdominal surgery, non-specific LBP  **Gender**  Not reported | NR | **Cost**  Long-term sick leave and work disability are costly for society and the individual, and assessment of work outcomes is needed to evaluate the effectiveness of health services.” (comment on Introduction)  […]” eHealth intervention aimed at improving return to work among sick-listed employees showed a positive cost-benefit for the involved stakeholders, but studies regarding the cost-effectiveness of eHealth interventions for work-related outcomes are few. eHealth interventions were initially developed as a tool for an interaction between health care professionals and patients.” (Comment introduction)  Two studies included cost in their outcomes (results) |
| 22 | Antunes et al (2019)[60]  SR  N=18 | ACI: acquired cerebral injury, SCI: spinal cord injury, ALS: amyotrophic lateral sclerosis    **Gender**  (Two studies included only men, one study included only women, and two studies included both sexes) | NR | **NR** |
| 23 | Marzano et al  (2017)[49]  SR  N=29 | Stroke, Multiple sclerosis, disability, pulmonary, cognition problems, Autism  **Gender**  NR | NR | **Cost**  Cost of technology mentioned as one of the barriers for older adult use. (results)  **Gender**  “The person's gender (males generally seem to have less difficulty in using computer applications.” (results) |
| 24 | Emmanouilidis et al (2021)[52]  SR  N=38 | Parkinson’s disease  **Gender**  Both sex included in all studies | NR | **NR** |
| 25 | Appleby et al (2019)[6]  SR  N=13 | Stroke  **Gender**  “Gender bias towards men (more male participants than women” | NR | **Age**  “It is unclear why there was an age-bias in the research literature on this topic. A possible explanation for excluding older people with stroke in telerehabilitation studies might be researchers’ perception that older people may experience discomfort or lack confidence with the use of technology and its impact on the effectiveness of telerehabilitation.” (results)  **Bias**  “Publication and language bias are likely and hence should be acknowledged.” (comment on limitations)  “ Given the search strategy was extensive and this resulted in seven publications from countries where English is not the first language means the impact of language bias has been minimised.” (comment on study limitations)  **Cost**  One study included cost as an outcome (results) |
| 26 | Geraedts et al. (2013)[35]  SR  N=32 | Cancer survivors, post CABG surgery, unilateral hip replacement, older adult with disability, intermittent claudication, heart failure, sedentary healthy older adult, cardiac rehabilitation, cardiac surgery, PD, lung patients, acute medical admission, older adult family caregiver, coronary artery disease, diabetes, seniors at risk for falls, women caring for relatives with dementia, diabetes, risk for falls, colorectal cancer survivors, male veterans, osteoporosis, peripheral neuropathy and THA  **Gender**  NR | NR | **Cost**  “More research was needed on feasibility and cost-effectiveness of the use of modern technology in home-based physical activity programmes for older adults.” (comments on Implications of the review for practice and research) |
| 27 | Amirova et al. (2021)[50]  MA  N=20 | Heart failure  **Gender**  “The majority of the sample was male 69.49%” | NR | **NR** |
| 28 | Bertolazzi et al (2023)[57]  Integrative Systematic review  N=14 | Chronic illness (heart failure, diabetes  **Gender**  Not reported | “Perceptions of the usefulness and ease of use of technology, as well as beliefs about privacy, may influence its adoption. While privacy concerns have not been observed for some technologies, such as telemedicine, they could be a barrier for other devices, including fall detection or bed occupancy sensors.” (background)  “Privacy seems to be a significant factor in hindering access to technology, especially when it comes to the use of the Internet for health information seeking and patient web portals. Since the ageing population tends to be unfamiliar with new technologies, training is a crucial factor in promoting and enhancing access to eHealth.” (results)  “Healthcare providers should consider introducing digital health devices to the elderly through personalised and easy-to-understand training. They should also reassure prospective users about privacy issues and provide constant and timely support in case of doubts or device malfunctions.” (comment on results) | **Cost**  **“**Several investigations consider the cost of technology as a barrier.” (results)  “To enhance access to health technologies, costs should be minimized or even eliminated, for example, by providing support to low-income individuals to access broadband.” (comments on discussion)  **Digital Literacy/education**  “Previous studies have found that older individuals with lower income and education levels have limited access to broadband, lower health literacy and lower digital competencies, which in turn leads to limited technology adoption.” (introduction)  “The impact of educational level on technology use appears to be more consistently supported by the literature. Well-educated older adults seem to have an advantage in adopting various technologies, whereas poor education limits their use and acceptance.” (results)  “Policymakers and technology developers should consider the needs of the most vulnerable and underprivileged social groups, particularly those with low household income and limited educational achievement.” (results) |
| 29 | Curry et al (2021)[36]  SR  N=8 | Lung Cancer  **Gender**  Not reported | NR | **Cost**  “Of the eight studies, none reported cost or financial cost of the study. Majority of the studies require health care professionals, researchers, and equipment, yet the monetary costs were not discussed. One study highlighted the absence of costing the intervention as a limitation.” (results)  “ The cost of travel is an out-of-pocket expensive which could be a barrier for people living with and beyond cancer to access appointments and treatments.” (comment on discussion) |
| 30 | Brick et al (2022)[37]  Scoping review  N=11 | Breast cancer  **Gender**  One study reported gender (60 females) | NR | **Ethnicity**  “The age of 60 years accounts for the median age of breast cancer diagnosis in black and white women and represents the majority of breast cancer diagnoses as well.” (comment on Introduction)  “ Yet, older Black populations experience similar breast cancer incidence rates as non-Hispanic white counterparts.” (comment) |
| 31 | Devi et al (2015)[22]  SR  N=8 | Coronary heart disease  **Gender**  In six studies, over 80% of participants were male and in five studies over 70% of participants were male | **Adverse events**  “No adverse effects of Internet‐based interventions have been observed in populations studied to date.” (comment results)  “An adverse intervention effect was reported in 1 trial.” (results) | **Cost**  “Two trials collected cost data: both reported that Internet‐delivered interventions are likely to be cost‐effective.” (introduction)  “Participant ethnicity: White (97.1%), black (1.0%), and other (1.9%).” (results) |
| 32 | Bostrom et al (2020)[46]  Narrative review  N=25 | Cardiac rehabilitation  **Gender**  In one study, 87% of the participants were male, and in another study, the majority were also male (82%). | **Safety**  “The recent HONOR trial, which evaluated an mHealth‐augmented home‐based exercise program for patients with PAD (in patients with a mean age of 70 years), also found no significant difference in significant adverse events between the intervention and control group. While further research is needed into the safety of home‐based CR programs, these results provide indirect support that they can be prescribed safely.” (comment on results) | **Digital literacy/ disabilities**  “Older adults have been slower to adopt newer technologies and devices than their younger counterparts.”(comment on results)  “Perceived difficulty of use has in fact been cited as a reason for lack of use of mHealth applications by older patients, but other barriers exist for example, age‐related sensory changes (fine motor skill deficiencies, vision loss) make certain devices more difficult to manipulate.” (comment on results)  “While literature supports that older adults may accept new technologies, they often do so with less confidence than younger adults. In a qualitative study on the use of mHealth in older patients with heart failure, lack of knowledge, and even “fear” of misusing the technology, were the most commonly cited barriers to mHealth adoption.” (comment on results) |
| 33 | Guan et al (2021)[38]  SR  N=28 | Cancer  **Gender**  Most of the participants were females | NR | **Ethnicity**  “Gender, Race and ethnicity reported. Most of the participants were Caucasians. With only two studies targeting parents of children and adolescents with cancer, the majority of the studies have focused on participants who were mostly female and White, and with an average age ranged from 44 to 72 years.” (results)  **Cost**  One study evaluated cost (results) |
| 34 | Chan et al (2021)[39]  SR &MA  N=31 | **Gender and morbidities**  NR | NR | **Ethnicity**  “Majority of the studies are conducted on healthy older adults, most of which were Caucasian and from developed countries. Thus, the results  may not be replicable in other countries with different ethnic and  economic backgrounds.” (results)  **Cost**  “e-interventions increases cost-effectiveness for patients by reducing travelling costs, consultation fees and loss of income from taking medical leave with no or little difference to health outcomes. After break-even from the initial set-up costs, telehealth is effective in cost-saving for healthcare in-situations.” (comment on results) |
| 35 | Beckie (2019)[16]  Narrative review  N=4 | Cardiovascular Disease  **Gender**  More than 65% males | NR | **Disability**  “Collectively, the studies of HBCR show little evidence of addressing the needs of disabled, frail, socially isolated or high-risk older adults with CVD. Before implementing an HBCR program for older adults, assessments of frailty, disability, comorbidity and, CVD risk are essential to providing patient-centered care. (comment on results)  **Cost**  “Access to a health professional through a web portal dashboard has the potential to be a low-cost strategy for fostering adherence to sustained healthy behavior changes. (comment on results)  **Digital literacy**  “Consideration of health and technology literacy as well as sensory and manual dexterity deficits among older adults that may interfere with optimal HBCR delivery is essential. “ (comment on results) |
| 36 | Bhattarai and Phillips (2017)[15]  Integrative review  N=9 | Pain: arthritic pain. Chronic pain, palliative care, oncology patients  **Gender**  NR | NR | **Cost**  “Older people are willing to learn and use digital technologies for pain management but experience some technological adoption barriers. One of the most highlighted barriers to use of digital technology was the concern relating to battery life (Parker et al., 2013), which resonates with earlier research. Future digital health technology interventions aimed at older people should consider implementation of cost-effective and power-efficient devices.” (Discussion)  **Digital Literacy**  Provision of device use training was a key facilitator. Unlike earlier reports that devices and programs need to be tailored as per older people’s need. More older people preferred to be device trained than having the devices tailored to their need. Given the high prevalence of cognitive impairment among older people and rapidly advancing field of technology, provision of ongoing training and support to older users should be considered when implementing digital technology-based intervention. An important facilitator supporting the adoption of digital technology is having close contact with clinicians and bi- directional flow of information. specially, as clinicians seem unprepared to deal with the large volumes of data generated by such interventions despite welcoming its use for pain management. " (Discussion) |
| 37 | Dequanter et al (2021)[23]  SR  N=72 | Cognitive Impairment  **Gender**  NR | **Adverse events**  “Outcome effects were labeled good if clinically or statistically significant intervention effects were reported or labeled as neutral or negative in case of no or adverse effects, respectively.” | **Cost**  “In order to make recommendations for their use in practice, other factors, such as their cost and acceptability, need to be considered. “ (discussion) |
| 38 | Allida et al (2020)[54]  SR & MA | Heart failure  **Gender**  NR | NR | **Cost**  “Only one study reported cost: "The mean costs per patient were €4,865 and €5,741 per quality-adjusted life years for heart failure management (HFM) website + usual care and usual care, respectively. The net-monetary benefit was positive (larger than 0) for HFM versus usual care". (results)  **Disabilities**  “There is a high prevalence of cognitive impairment in older people living with heart failure. It is crucial that future educational interventions include screening for cognitive impairment and focus on optimising care and outcomes in individuals living with both HF and cognitive impairment. “(Discussion) |
| 39 | Xie et al (2021)[55]  SR & MA  N=6 | Osteoarthritis  **Gender**  Male sand females included in all studies | NR | NR |
| 40 | Choukou et al (2021)[26]  Scoping review  N=26 | Chronic diseases  **Gender**  Not reported | **Respect and knowledge of cultural diversity**  “to date, the development of telehealth technologies has  seldom considered the important and unique cultural factors of Indigenous populations, as cultural appropriateness is one element influencing acceptance of use.”  “Cultural icons are used as virtual rewards when Participants achieved their goals.”  “Apps contained culturally relevant information, such as about activity groups specifically for a given community,  promotion of wellbeing through spiritual concepts, customs, blessings, culturally relevant recipes, proverbs, and culturally  tailored motivational messages.” | **Cost**  “Seven studies, including an economic  evaluation paper, unanimously concluded that telehealth solutions allow for cost savings by minimising the need to travel and decreasing the burden on budgets of central clinics.” (results)  **Ethnicity**  Indigenous from different countries (results)  **Fair access**  “to date, the development of telehealth technologies has  seldom considered the important and unique cultural factors of Indigenous populations, as cultural appropriateness is one element influencing acceptance of use.” (introduction) |
| 41 | Gaspar and Lapão (2021)[43]  SR  N=21 | Balance disorders  **Gender**  “Most of the studies included more women than men. In four studies, only women participated. The decision to only recruit women was explained in one study as “to avoid the influence of gender differences on risk of falling”. Two studies excluded the few male participants, and the remaining article did not describe the reason for the exclusive participation of women. One study did not describe the age range or the gender distribution of the participants.” | **Safety**  “WiiFit feasible to safely use, Ski Slalom game similar effect as computerized dynamic posturography.” | **Education/Digital Literacy**  "None of the studies explored the previous health literacy of the participants. Only two papers described the educational level of the participants. we observed a constraint related to the use of devices that are not fully adequate to match the abilities of elderly people.“ (results)  **Cost**  “Balance disorders and consequent falls have progressively represented a burden of disease, accompanied by high costs and pressure on the social services and health care systems related to medical care.” (comments on background)  “The cost was estimated at about US $768 per episode, translating to an annual national cost of US $757 million. In the same context, cardiovascular diseases (linked to 16.5% of these episodes) represented a cost of approximately US $1489 per episode for an annual cost of US $941 million. By comparison, cerebrovascular diseases only accounted for 3.1% of these episodes, but with a cost per episode of approximately US $1059 or an annual cost of US $127 million. With the progressive aging of the population, worsening of this situation is expected in the future [25]. Indeed, vertigo is already contributing to the increasing trend of health care costs, which is linked to the aging of the population.” (comments on background)  “The use of eHealth seems to decrease costs associated with both institutionalization and unnecessary hospital visit.” (comments on background) |
| 42 | Alaimo et al (2021)[56]  SR  N=8 | Cognitive problems and healthy older adults  **Gender**  Not reported | NR | **Education**  Reported (Mean of education: 5.8y-18y) (results) |
| 43 | Reeder et al (2016)[20]  Narrative review  N=9 | "Mobility-impaired community-dwelling older adults; Balance-impaired older adults; post-stroke survivors with hand paralysis; Head and neck cancer surgery patients  Pre-admission orthopedic surgery patients; Physically inactive older adults;  Total knee arthroplasty patients; post-stroke survivors; Total knee arthroplasty patients"  **Gender**  Not reported | **Safety**  “Creating opportunities for more effective therapies and minimizing adverse health events is essential.” | **Cost**  “In an era of decreasing financial resources for patient care and increased constraints to health care reimbursement, maximizing patient adherence, creating opportunities for more effective therapies, and minimizing adverse health events is essential.” (comments discussion) |
| 44 | Ambrens et al (2022) [24]  SR & MA  N=14 | **Gender**  “Both males and females were included in 11 studies, all had a higher percentage of female participants. Two studies included only female participants, one study included only male participants.” | **Adverse event**  “Also extracted were data on drop out, attrition, adverse events and intervention features such as implementation fidelity, evidenced-based theory, tailoring, supervision, intervention acceptability.” (methods)  “Eight included studies (57%) measured adverse events. Of those reported, no major adverse events were related to the intervention.” (results) | **Cost**  “Falls place a significant burden on health systems and can result in serious long-term costs to the individual.” (introduction)  “Future research needs to explore the long-term impact, cost-effectiveness and sustainability of eHealth-delivered programmes on balance and fall-related outcomes in older people living in the community.” (Discussion) |
| 45 | Solis-Navarro et al (2022)[31]  SR & MA  N=26 | Cardiorespiratory problems, balance disorders, musculoskeletal disorders, cancer and others.  **Gender**  65% females | **Safety**  “Current recommendations indicate the importance of  training lower limb strength in older adults, making it a very frequently training task, safe to perform without direct  supervision and easy to progress (overload).” (discussion) | NR |
| 46 | Del Pino et al (2022) [47]  SR  N=8 | Cardiological diseases  **Gender**  Not reported | Adverse events  “However, adverse events were higher during treatment in the telerehabilitation group.” (discussion) | **Access**  “Telerehabilitation could expand the access to perform rehabilitation for people that could not have access to traditional clinic care either for personal, geographical, economic reasons or due to the public health system.” (comments on strengths and limitations)  **Cost**  “Three studies were related to the costs and results of telerehabilitation in neurological diseases (specifically in stroke).”  “The studies included cost-analysis, cost-benefit, cost-effectiveness, or cost-utility. In total, four studies found significant cost/savings per person between $565.66 and $2,352.00 (p < 0.05). In contrast, most studies found differences in costs and clinical effects between the telerehabilitation performed and the rehabilitation performed at the clinic. Just one study found quality-adjusted life years (QALY) significant differences between groups [Incremental cost-effectiveness ratio (ICER) per QALY ($−21,666.41/QALY).” (results)  “Telerehabilitation seems to be as clinical and cost-effective as traditional rehabilitation, even if, generally, telerehabilitation is less costly. More research is needed to evaluate health-related quality of life and cost-effectiveness in other neurological diseases.” (discussion)  “The systematic review revealed that just eight studies focused on the costs and effectiveness of neurological and cardiological telerehabilitation. According to the studies reviewed, telerehabilitation was more cost-effective than traditional rehabilitation at the clinic. Half of the studies found significant differences in cost/savings per person between the telerehabilitation performed and the traditional one at the clinic.” (discussion)  “It is highlighted that in order to make proper clinical decisions and decide whether a new telerehabilitation program is good enough, from the clinical and economic perspective, to be implemented in the public or private health system, cost needs to be considered, and therefore, cost-effectiveness analyses are needed.” (results) |
| 47 | Bacanoiu and Danoiu (2022)[40]  SR  N=34 | Healthy older adults and elderly with motor and cognitive impairments from Parkinson’s disease and Alzheimer’s disease  **Gender**  “All randomized studies used in the synthesis included a large number of individuals of both sexes.” | NR | **Socioeconomic status (SES)**  “Older adults living in disadvantaged areas, dwelling communities, discharge hospitalization, or housing care, for whom travel to specialists would be difficult due to financial expensive; and there are limitations on the costs of instruments/tools used or the extend periods of time allocated to effective monitoring of remote older adults by physicians and care givers. To these is added the incorrect assessment of the needs of the elderly and the degree of dependency, which makes the interventions only formal.” (comment on conclusion)  **Geographic location/SES**  “Rural population included in one study. The need for the future is to implement programs to promote health and healthy diet for the elderly in both urban and rural areas, regardless of their income and, especially, to combat poor socio-economic status. In this sense, health policies will have to be approached to promote a functional diet in both rural and urban areas, but also financial support for those who benefit from low incomes, who cannot provide a healthy diet.” (results) |
| 48 | Zhang et al (2022)[41]  Scoping review  N=39 | Hip fracture  **Gender**  Not reported | NR | **Geographic location**  “A total of 31 studies were conducted in high income countries, with the remaining 8 studies in low- and middle-income countries (LMICs).” (results) |
| 49 | Ding et al (2023)[25]  Scoping review  N=20 | Stroke, Parkinson's disease, hip fracture, special diseases, cognitive impairment, obstructive pulmonary disease, acquired brain injury, cancer, manual wheelchair users, older adults  **Gender**  Not reported | **Adverse events**  “No serious adverse events were reported in any of the studies we included, so the safety of telehealth OT can be initially confirmed.” (discussion) | **Access**  “The majority of older people with functional impairments have no access to timely occupational interventions because they have limited traditional rehabilitation resources. (Introduction)  **Cost**  “Although the COVID-19 pandemic has seriously further affected patients’ access to rehabilitation, telehealth is an attractive alternative to in-person rehabilitation. older people have difficulty adhering to traditional rehabilitation because of its high cost, the time spent travelling between rehabilitation facilities and their home, and the safety issues that result from the journey.” (results) |
